# “Tasting” fructose with pancreatic beta-cells: modulation of insulin release by sweet taste receptor signaling and its role in metabolic diseases

**DOI:** 10.1186/1753-6561-6-S3-P29

**Published:** 2012-06-01

**Authors:** George A Kyriazis, Kathleen R Mosure, Mangala M Soundarapandian, Richard E Pratley, Björn Tyrberg

**Affiliations:** 1 Diabetes & Obesity Research Center, Sanford-Burnham Medical Research institute, Orlando, FL 32827, USA; 2 Translational Research Institute for Metabolism and Diabetes, Florida Hospital, Orlando, FL 32804, USA

## Background

Although glucose is indispensable for the stimulation of insulin release, numerous other insulin secretagogues have been identified. For instance, the dietary monosaccharide fructose potentiates insulin secretion *in vitro*, but the mechanism and physiological significance remains unclear. The T1R2-T1R3 heterodimer of G protein-coupled receptors mediates sweet sensing in the tongue and ablation of either subunit obliterates sweet taste. We hypothesized that the effects of fructose on insulin release may be mediated by sweet taste receptors (TRs) on beta-cells.

## Materials and methods

Mice with the homozygous deletion for the T1R2, T1R3, or TRPM5 gene (Dr. Zuker, Columbia University) were bred and genotyped in-house. *In vitro* static insulin release (ELISA, Mercodia) was assessed using cultured human and mouse islets incubated in custom-made wells with various treatments. Fura-2 based calcium imaging was performed using MIN6 cells or dispersed mouse beta-cells. Phospholipase C (PLC) activation was measured using Total Internal Reflection (TIRF) Microscopy in transfected MIN6 cells with a PH_PLCδ1_-GFP construct. In *vivo* experiments were performed with 8-10 week old catheterized conscious male mice on regular chow after 5-hour fasting.

## Results

Human and mouse islets express sweet TRs. Fructose (10.0mM) rapidly activated PLC and increased intracellular calcium (Ca^2+^_i_) and insulin release at 8.3mM glucose in wild type (WT) islets and MIN6 cells, but these effects were absent in T1R2 knockout (T1R2^-/-^) islets. Similar to mouse islets, fructose stimulated insulin release in human islets and these effects were blocked by lactisole, a human-specific inhibitor of T1R3. *In vivo*, an intravenous bolus of fructose (1.0g/kg) rapidly increased plasma insulin in WT, but not in T1R2^-/-^ mice. Glucose-stimulated insulin release (GSIS) in WT mice was potentiated by low physiological concentrations of fructose (3.0mM *in vitro*; 0.3g/kg *in vivo*), but these effects were absent in T1R2^-/-^ mice. The transient receptor potential channel M5 (TRPM5) mediates TR signaling in the tongue, contributing to cell membrane depolarization. Islets from TRPM5 knockout mice (TRPM5^-/-^) failed to increase Ca^2+^ and insulin release in response to fructose. Finally, the expression of TRs is reduced in islets of diabetic mouse models (db/db) and is associated with impaired fructose-induced insulin release.

## Conclusions

Fructose is a natural ligand for functional sweet TRs expressed on mouse and human beta-cells. Pancreatic taste receptors sense circulating fructose and activate a distinct signaling pathway involving PLC, TRPM5 and Ca^2+^_I_ influx that potentiates GSIS [1]. Our data together with previous reports showing that sweet TRs in the intestinal epithelium stimulate dietary glucose absorption and GLP-1 secretion, suggest a novel TR-dependent intestinopancreatic axis that participates in the regulation of postprandial insulin release by absorbed sugars. These data suggest a potential link between high-fructose consumption and the development of adverse metabolic effects. Interestingly, beta-cell TR expression and function is reduced in diabetic mouse phenotypes, also suggesting that impaired TR signaling may play a role in the pathogenesis of metabolic diseases.
